# Reviewer Acknowledgement 2013

**DOI:** 10.1186/1745-6215-15-21

**Published:** 2014-01-21

**Authors:** Daniel Shanahan

**Affiliations:** 1 BioMed Central, 236 Gray's Inn Road, WC1X 8HB, London, United Kingdom

## Abstract

**Contributing reviewers:**

A peer-reviewed journal would not survive without the generous time and insightful comments of the reviewers, whose efforts often go unrecognized. *Trials* has been blessed by the support of highly-qualified peer reviewers, and the Editors-in-Chief, Doug Altman, Curt Furberg and Jeremy Grimshaw, and staff of the journal would like to show their appreciation by thanking the following for their invaluable assistance with review of manuscripts for the journal in Volume 14 (2013).

## 

Purva Abhyankar

United Kingdom

Christopher Adlbrecht

Austria

Lawrence Afrin

United States of America

Ritesh Agarwal

India

Rakesh Aggarwal

India

Noori Akhtar-Danesh

Canada

Ather Ali

United States of America

Kelli Allen

United States of America

Pablo Alonso-Coello

Spain

Rustam Al-Shahi Salman

United Kingdom

Fernando Althabe

Argentina

Ruth A. Anderson

United States of America

Susan Anenberg

United States of America

Stavros Antoniou

Germany

Sabina Antoniu

Romania

Vigneswari Aravindalochanan

India

Carola Arfeller

Germany

Rebecca Armstrong

Australia

Arnoud Arntz

Netherlands

Bruce Arroll

New Zealand

Arne Astrup

Denmark

Max Oscar Bachmann

United Kingdom

Sean Bagshaw

Canada

Nicolas Bakinde

United States of America

Olaf Bakker

Netherlands

Liu Baoyan

China

William Barnett

United States of America

Meagan Barry

United States of America

Ivan Bautmans

Belgium

Derrick Bennett

United Kingdom

Louise Berendt

Denmark

Jesse Berlin

United States of America

Wanderley Bernardo

Brazil

Jean-Marie Berthelot

France

Joseph Beyene

Canada

Sanjay Bhananker

United States of America

Mohit Bhandari

Canada

Aneel Bhangu

United Kingdom

Zhaoxiang Bian

Hong Kong

Lydia Bird

United Kingdom

Felicity Bishop

United Kingdom

Delia Boccia

United Kingdom

Davide Bolignano

Italy

Christine Bond

United Kingdom

Jonathan Boote

United Kingdom

Bijan Borah

United States of America

John Joseph Borg

Malta

Josée Bouchard

Canada

John Bourke

United Kingdom

Koen Boussery

Belgium

Isabelle Boutron

France

Angela Bowen

Canada

Deborah Bowen

United States of America

Peter Bower

United Kingdom

Ursula Bowler

United Kingdom

Rollin Brant

Canada

Daniel Bratton

United Kingdom

Darren Brenner

United States of America

Oana Brosteanu

Germany

Richard Brown

United States of America

Joshua Burns

Australia

Katie Buston

United Kingdom

Stephen Butler

United Kingdom

Tracey Bywater

United Kingdom

Philip Calder

United Kingdom

David Calhoun

United States of America

Robert Califf

United States of America

Melainie Cameron

Australia

Michael Camilleri

United States of America

Michael Campbell

United Kingdom

Waldemar (Wally) Carlo

United States of America

Giselle Carnaby

United States of America

Andrew Carr

United Kingdom

Daniel Carr

United States of America

Christopher Carroll

United Kingdom

Greg Carter

Australia

Rickey Carter

United States of America

Kate Cavanagh

United Kingdom

Barbara Chadwick

United Kingdom

An-Wen Chan

Canada

Sally Chan

Singapore

Jackie Chandler

United Kingdom

Luis Enrique Chaparro

Colombia

Georgina Charlesworth

United Kingdom

Meng-Hua Chen

China

Chi Wai Cheung

Hong Kong

Dennis Chi

United States of America

Wai Tong Chien

Hong Kong

Mai Jeanette Maidy Chinapaw

Netherlands

Sun-Mi Choi

South Korea

Maud-Christine Chouinard

Canada

Rong Chu

Canada

Joseph Ciarrochi

Australia

Renata Cifkova

Czech Republic

Laura Clark

United Kingdom

Jan Clarkson

United Kingdom

Marie Cleary

United Kingdom

Erik Cobo

Spain

Jennifer Coghlan

Australia

David Cohen

United Kingdom

Judith Cohen

United Kingdom

Alain Cohen Solal

France

Clare Collins

Australia

Philip Conaghan

United Kingdom

Hari Conjeevaram

United States of America

Jonathan Alistair Cook

United Kingdom

Cindy Cooper

United Kingdom

Simon Coulton

United Kingdom

Brian Cox

New Zealand

Susan Cox

Canada

Lisa Coyne

United States of America

Joseph Cravero

United States of America

Jack Cuzick

United Kingdom

Ann Dadich

Australia

Lars Dahlstrom

Sweden

Helen Dakin

United Kingdom

Gayle Dakof

United States of America

Sarah Damery

United Kingdom

Margie Danchin

Australia

Julie Darbyshire

United Kingdom

Harry J. De Koning

Netherlands

Christian De Mey

Germany

Mary De Vera

Canada

Olivia Dean

Australia

Sai Krishna Degala

India

Rob Den Otter

Netherlands

Sarah Denford

United Kingdom

Suzanne Denieffe

Ireland

Colin Derdeyn

United States of America

Andrea Deregibus

Italy

Niveditha Devasenapathy

India

John Devlin

United States of America

Sean Dinneen

Ireland

Dennis Dixon

United States of America

Stephan Dombrowski

United Kingdom

Tara Donker

Australia

J. Peter Donnelly

Netherlands

Allan Donner

Canada

D. John Doyle

United States of America

Tomas Drabek

United States of America

Klaus Drieschner

Netherlands

Barney Dunn

United Kingdom

Graham Dunn

United Kingdom

Laura Dunn

United States of America

Sourabh Dutta

India

Louise Dye

United Kingdom

Diana Eccles

United Kingdom

Donald Edmondson

United States of America

Phil Edwards

United Kingdom

Sarah Edwards

United Kingdom

Matt Egan

United Kingdom

Hannelore Ehrenreich

Germany

Ole Marius Ekeberg

Norway

Inger Ekman

Sweden

Charles Elder

United States of America

Sandra Eldridge

United Kingdom

Susan Ellenberg

United States of America

Tom Elliott

United Kingdom

David Engler

United States of America

Perenlei Enkhbaatar

United States of America

Jianqiao Fang

China

Jian Farhadi

United Kingdom

Morag Farquhar

United Kingdom

Carlos Alberto Feldens

Brazil

Dean Fergusson

Canada

Ricardo Fernandes

Portugal

Roberto Ferrari

Italy

Sarah Fidler

United Kingdom

Shona Fielding

United Kingdom

Per Fink

Denmark

Edwin Fisher

United States of America

John Fleming

United States of America

Thomas Fleming

United States of America

Benjamin Fletcher

United Kingdom

Irene Floriani

Italy

Robert Folmer

United States of America

Tobias Freund

Germany

Lawrence Friedman

United States of America

Flavio Fuchs

Brazil

Anne-Sofie Furberg

Norway

Ivana Furimsky

Canada

Toshiaki Furukawa

Japan

Pedro Gamito

Portugal

Simon Gates

United Kingdom

Judith L Gellatly

United Kingdom

Stacie Geller

United States of America

Jochen Gensichen

Germany

Lynn Gerald

United States of America

Shahyar Gharacholou

United States of America

Uday Ghoshal

India

Thijs Giezen

Netherlands

Aleksandra Gilis-Januszewska

Poland

Donna Gillies

Australia

Timothy Girard

United States of America

Nathalie Godart

France

Lutz Goldbeck

Germany

Charlie Goldsmith

Canada

Manuel Gomes

United Kingdom

Mithat Gonen

United States of America

Jayashree Gopal

India

Michael Gordon

Canada

Tommaso Gori

Germany

Irene Gottlob

United Kingdom

Stephen Graham

Australia

Aileen Grant

United Kingdom

Xavier Griffin

United Kingdom

Ellie Grossman

United States of America

Miriam Grover

United Kingdom

Elizabeth Grunfeld

United Kingdom

Francisco Gude

Spain

François Gueyffier

France

Freedom Nkhululeko Gumedze

South Africa

Clement Gwede

United States of America

Marta Gwinn

United States of America

Michael Hamblin

United States of America

Freddie Hamdy

United Kingdom

Richard Hammerschlag

United States of America

Ji-Sheng Han

China

Andria Hanbury

United Kingdom

Lesley-Ann Hanson-Bellefeuille

Canada

James Hargreaves

United Kingdom

Kathleen Harrington

United States of America

Ian Harris

Australia

Mark Harrison

United Kingdom

Rosamund Harrison

Canada

Markus Hartmann

Germany

David Healy

United States of America

Nick Heather

United Kingdom

Hiddo Heerspink

Netherlands

Ilkka Helanterä

Finland

Peter Herbison

New Zealand

Sharlene Hesse-Biber

United States of America

Philip Hill

New Zealand

Trina Hinkley

Australia

Oliver Hirsch

Germany

Zoe Hoare

United Kingdom

Tammy Hoffmann

Australia

Celia Holland

Ireland

Kam-Lun Ellis Hon

Hong Kong

Hideo Honda

Japan

Pieter Honkoop

Netherlands

Val Hopwood

United Kingdom

Louise Michele Howard

United Kingdom

Asbjørn Hróbjartsson

Denmark

Jui-Chien Hsieh

Taiwan

Sally Hunsberger

United States of America

Michael Hurlburt

United States of America

Stephen Hwang

Canada

Sayeed Ikramuddin

United States of America

Marie Christine Iliou

France

Kjetil Isaksen

Norway

Khalida Ismail

United Kingdom

Lewis James

United Kingdom

Bo-Hyoung Jang

South Korea

Muralidharan Jayashree

India

Masahito Jimbo

United States of America

Moyez Jiwa

Australia

Kent Johnson

Australia

Carys Jones

United Kingdom

Christina Jones

United Kingdom

Andrew Judge

United Kingdom

Andrew Jull

New Zealand

Ed Juszczak

United Kingdom

Juliana Kain

Chile

Norma Kanarek

United States of America

Thanos Karatzias

United Kingdom

Amol Karmarkar

United States of America

Morten Karsdal

Denmark

Adnan Kastrati

Germany

Brian Kavanagh

Canada

Steven Kelder

United States of America

Timothy Kenealy

New Zealand

David Kent

United States of America

Ngaire Kerse

New Zealand

David Kessler

United Kingdom

Michele Kiely

United States of America

Yongjoo Kim

South Korea

Young Tae Kim

South Korea

Ann Louise Kinmonth

United Kingdom

Rosemary Knight

United Kingdom

Felix Koepcke

Germany

Jeff Kraut

United States of America

Michael Krawinkel

Germany

Catherine Kreatsoulas

United States of America

Ian Kronish

United States of America

Karen Kuntz

United States of America

Edmund La Gamma

United States of America

Jacques Lacroix

Canada

Max Lafontan

France

Robert Landewe

Netherlands

Deirdre Lane

United Kingdom

Lillebeth Larun

Norway

Marissa Lassere

Australia

Dimitrios Lathyris

Greece

Romy Lauche

Germany

Jim Lavery

Canada

Able Lawrence

India

Julia Lawton

United Kingdom

Dominique Le Guludec

France

Claire Leathem

United Kingdom

Andrew Leaver

Australia

Anna Lee

Hong Kong

Hyangsook Lee

South Korea

Juneyoung Lee

South Korea

Myeong Soo Lee

South Korea

Pearl Lee

United States of America

Wilma Leslie

United Kingdom

Liran Levin

Israel

George Lewith

United Kingdom

Joel Lexchin

Canada

Ai-Hsien Li

Taiwan

Ai-Hsien Li

Canada

Fan-Rong Liang

China

Zhaohui Liang

China

Charles Lidz

United States of America

Hung-Chih Lin

Taiwan

Klaus Linde

Germany

Kristen Linton

United States of America

Ingrid Liodden

Norway

Alyson Littman

United States of America

Jianping Liu

China

Sylvie Lo Fo Wong

Netherlands

Lynne Lohfeld

Canada

Carl Lombard

South Africa

Melva Louisa

Indonesia

Aiping Lu

China

Thomas Lumley

United States of America

Jinhui Ma

Canada

Manfred Müller

Germany

R. Loch MacDonald

Canada

Graeme MacLennan

United Kingdom

Hugh MacPherson

United Kingdom

Chris Maher

Australia

Mohandas Mallath

India

Ranjit Manchanda

United Kingdom

Antonio Mantovani

United States of America

Christine Manyando

Zambia

Jun Mao

United States of America

Klaus Martiny

Denmark

Serge Masson

Italy

Nancy Mayo

Canada

Lawrence Mbuagbaw

Cameroon

Judith McFarlane

United States of America

Richard McGee

Australia

Sharon McKinley

Australia

Brian McKinstry

United Kingdom

Mary McMurran

United Kingdom

Roseanne McNamee

United Kingdom

Andrew McRae

Canada

Noreen Mdege

United Kingdom

Usha Menon

United Kingdom

Martin Meremikwu

Nigeria

Sally Merry

New Zealand

James Meschia

United States of America

George Mgomella

United Kingdom

Christoph Michalski

Germany

Cathy Mihalopoulos

Australia

José C. Millán-Calenti

Spain

Nicola Mills

United Kingdom

Geltrude Mingrone

Italy

David Mitchell

United Kingdom

Alon Mogilner

United States of America

Andrew Moore

United Kingdom

James Moriarty

United States of America

William Mortenson

Canada

Yoshiharu Motoo

Japan

Gail Anne Mountain

United Kingdom

Brendan Mulhern

United Kingdom

Michael Mullen

United States of America

Manuel Muñoz

Spain

Helen Murphy

United Kingdom

Gordon Murray

United Kingdom

Meredith Nahm

United States of America

Luigi Naldi

Italy

Eve Namisango

Uganda

Martha Nason

United States of America

Mona Nasser

United Kingdom

Bruce Neal

Australia

Laurent Nicod

Switzerland

Merete Nordentoft

Denmark

John Norrie

United Kingdom

Paulina Nowicka

Sweden

Francine Ntoumi

Congo

Xavier Nuñez

Spain

Bright Nwaru

Finland

Jael Obiero

Kenya

Mark Andrew O'Connor

Australia

Rory O'Connor

United Kingdom

Martin Orrell

United Kingdom

David Osborn

Australia

Natalia Oudovenko

Spain

Ana Palacio

United States of America

Stefano Palomba

Italy

Shangling Pan

China

Jongbae Park

United States of America

Michael Parker

United Kingdom

Shefton Parker

Australia

Nick Parsons

United Kingdom

Gerard Pasterkamp

Netherlands

Janet Peacock

United Kingdom

Roger Pearson

Ethiopia

Claudio Pedone

Italy

Michael Peeters

United States of America

Sean Perrin

United Kingdom

Tim Peters

United Kingdom

Tim Peto

United Kingdom

Waltraud Pfeilschifter

Germany

K. Luan Phan

United States of America

Christopher Phillips

United States of America

Patrick Phillips

United Kingdom

Penelope Phillips-Howard

United Kingdom

Ruth Pickering

United Kingdom

Marie Pitkethly

United Kingdom

Bertram Pitt

United States of America

Robert Platt

Canada

Jenny Ploeg

Canada

Janice Pogue

Canada

Riccardo Polosa

Italy

Raphael Porcher

France

Andrew Prayle

United Kingdom

Peter Proff

Germany

Audrey Prost

United Kingdom

Eleanor Pullenayegum

Canada

Duncan Raistrick

United Kingdom

Susan Ramlo

United States of America

Tim Ramsay

Canada

Lars Rejnmark

Denmark

Clare Relton

United Kingdom

Ludovic Reveiz

United States of America

Rachel Richesson

United States of America

Fernando Rico-Villademoros

Spain

Lynn Riddell

Australia

Nola Ries

Canada

David Riley

United States of America

Se-Joong Rim

South Korea

Chris Roberts

United Kingdom

Ulrike Rothe

Germany

Marta Rubiera

Spain

Clare Rutterford

United Kingdom

Jo Rycroft-Malone

United Kingdom

Everardo Saad

Brazil

Zainab Samaan

Canada

Helen Sammons

United Kingdom

Peter Sandercock

United Kingdom

Elisabeth Sanders

Netherlands

Eugenio Santoro

Italy

Carlos Santos-Gallego

United States of America

Daniel Sargent

United States of America

Jeffrey Saver

United States of America

Roberta Scherer

United States of America

Gregory Schilero

United States of America

Felix Schlachetzki

Germany

Christopher Schmickl

United States of America

Rosa Schnyer

United States of America

Andrew Scholey

Australia

David Schriger

United States of America

Sven Schroeder

Germany

Anneke Schroen

United States of America

Wilhelm Schulte-Mattler

Germany

Russell Schutt

United States of America

Matthias Schwannauer

United Kingdom

Peter Schwarz

Germany

David Scott

United Kingdom

Grant Searchfield

New Zealand

David Seder

United States of America

Arnaud Seigneurin

France

Larissa Shamseer

Canada

Rajiv Sharma

United States of America

Bill Shaw

United Kingdom

Valerie Shilling

United Kingdom

Byung-Cheul Shin

South Korea

Heidi Siddle

United Kingdom

Derrick Silove

Australia

Umberto Simeoni

France

Alan Simpson

United Kingdom

Lara Simpson

United States of America

Aloysius Niroshan Siriwardena

United Kingdom

Daniel Sjoberg

United States of America

Elizabeth Skinner

Australia

Roman Skoracki

United States of America

Patrick Smith

United Kingdom

Jaapjan Snoep

Netherlands

Claire Snowdon

United Kingdom

Harald Sourij

United Kingdom

Sophie Staniszewska

United Kingdom

Lindsay Stead

United Kingdom

Justin Stebbing

United Kingdom

L. Stein

United States of America

Holger Stenzhorn

Germany

Robert Stevens

United States of America

Catherine Stewart

United Kingdom

Kay Stewart

Australia

Lynne Stobbart

United Kingdom

Katie Stone

United States of America

Nancy Suchman

United States of America

Bela Suki

United States of America

Eva Szabo

United States of America

Shahrad Taheri

United Kingdom

Betty Tai

United States of America

Alan Tait

United States of America

Monica Taljaard

Canada

Tyna Taskila

United Kingdom

David Taylor

United Kingdom

Lehana Thabane

Canada

Puvan Tharmanathan

United Kingdom

Simon Thomson

United Kingdom

Vittoria Tibaldi

Italy

Agnes Tiwari

China

Rebecca Tooher

Australia

David J Torgerson

United Kingdom

Ferran Torres

Spain

Shaun Treweek

United Kingdom

Yun-Fang Tsai

Taiwan

Sarah Tschudin Sutter

Switzerland

Tony Tse

United States of America

Komla Tsey

Australia

Martin Turner

Australia

Flora Tzelepis

Australia

Dirk Ubbink

Netherlands

Seshiah V.

India

Lisette Van Den Bemt

Netherlands

Aleid Van Wassenaer-Leemhuis

Netherlands

Satyanarayana Vedula

United States of America

Galina Velikova

United Kingdom

Andrew Vickers

United States of America

Marcos Vidal Melo

United States of America

Vasco Videira Dias

Portugal

Vijay Viswanathan

India

Vladimir Vuksan

Canada

Rolf Wahlstrom

Sweden

Stephen John Walters

United Kingdom

Yongjun Wang

China

Joanna Wardlaw

United Kingdom

Martin O. Weickert

United Kingdom

Charles Weijer

Canada

Stephanie Shifra Weinreich

Netherlands

Christopher Weir

United Kingdom

Robert Weissert

Germany

Dave Wendler

United States of America

Chunhua Weng

United States of America

Gesine Weser

Germany

Elisabeth Westerdahl

Sweden

Keith Wheatley

United Kingdom

Phil Wiffen

United Kingdom

Ian Wilkinson

United Kingdom

Hywel Williams

United Kingdom

Andrew Wilson

United Kingdom

Ed Wilson

United Kingdom

Davinia Withington

Canada

Barbara Wizner

Poland

Cheng-Hsien Wu

Taiwan

Longyang Wu

Canada

Taixiang Wu

China

Boxi Yan

China

Jieyun Yin

United States of America

Tae-Han Yook

South Korea

Mitsuyoshi Yoshida

Japan

Bridget Young

United Kingdom

Hayley Young

United Kingdom

Flora Zagouri

Greece

Xiaoxi Zhang

United States of America

Guo-Qing Zheng

China

Merrick Zwarenstein

Canada

